# Detection of *ESR1* Mutations in Primary Tumors and Plasma Cell-Free DNA in High-Grade Serous Ovarian Carcinoma Patients

**DOI:** 10.3390/cancers14153790

**Published:** 2022-08-04

**Authors:** Dimitra Stergiopoulou, Athina Markou, Lydia Giannopoulou, Paul Buderath, Ioanna Balgkouranidou, Nikolaos Xenidis, Stylianos Kakolyris, Sabine Kasimir-Bauer, Evi Lianidou

**Affiliations:** 1 Analysis of Circulating Tumor Cells Lab, Laboratory of Analytical Chemistry, Department of Chemistry, University of Athens, 15771 Athens, Greece; 2 Department of Gynecology and Obstetrics, University Hospital of Essen, University of Duisburg-Essen, 45359 Essen, Germany; 3 Department of Oncology, Medical School, Democritus University of Thrace, 25510 Alexandroupolis, Greece

**Keywords:** liquid biopsy, cell-free DNA, cfDNA, circulating tumor DNA, ctDNA, *ESR1* mutations, ovarian cancer, droplet digital PCR, drop-off ddPCR

## Abstract

**Simple Summary:**

In the present study we evaluated the frequency and the clinical relevance of *ESR1* mutations in high-grade serous ovarian cancer (HGSOC). Drop-off droplet digital PCR (ddPCR) was first used to screen for *ESR1* mutations in primary tumors (formalin-fixed paraffin-embedded, FFPEs) from HGSOC patients and plasma cell-free DNA (cfDNA) samples from advanced and metastatic ovarian cancer patients. We further used the recently developed *ESR1*-NAPA assay to detect individual *ESR1* mutations in drop-off ddPCR-positive samples. We report for the first time the presence of *ESR1* mutations in 15% of FFPEs and in 13.8% of plasma cfDNA samples from advanced and metastatic ovarian cancer patients.

**Abstract:**

*ESR1* mutations have been recently associated with resistance to endocrine therapy in metastatic breast cancer and their detection has led to the development and current evaluation of novel, highly promising therapeutic strategies. In ovarian cancer there have been just a few reports on the presence of *ESR1* mutations. The aim of our study was to evaluate the frequency and the clinical relevance of *ESR1* mutations in high-grade serous ovarian cancer (HGSOC). Drop-off droplet digital PCR (ddPCR) was first used to screen for *ESR1* mutations in 60 primary tumors (FFPEs) from HGSOC patients and in 80 plasma cell-free DNA (cfDNA) samples from advanced and metastatic ovarian cancer patients. We further used our recently developed *ESR1*-NAPA assay to identify individual *ESR1* mutations in drop-off ddPCR-positive samples. We report for the first time the presence of *ESR1* mutations in 15% of FFPEs and in 13.8% of plasma cfDNA samples from advanced and metastatic ovarian cancer patients. To define the clinical significance of this finding, our results should be further validated in a large and well-defined cohort of ovarian cancer patients.

## 1. Introduction

Ovarian cancer remains the cancer with the worst survival rates in women, as in most cases it is diagnosed at an advanced stage [1]. It is the second most frequent malignancy, following breast cancer, in women over the age of 40, especially in developed countries [2]. For primary disease, the customary treatment is debulking surgery accompanied by first-line platinum and paclitaxel-based chemotherapy [1]. The majority of patients respond to primary treatment but more than half of them will acquire chemo-resistance and consequently recurrent disease [1]. Epithelial ovarian cancer is the most frequent type, with histological and molecular heterogeneity [1,2]. Serous tumors are classified into high-grade serous carcinomas (HGSCs) and low-grade serous carcinomas (LGSCs) [2]. The former is a highly aggressive disease that is often diagnosed at an advanced FIGO (International Federation of Gynecology and Obstetrics) stage [1]. Overall survival (OS) remains low, even though there have been slight improvements in therapy [1]. There are a few available targeted therapies for ovarian cancer, such us the anti-angiogenetic antibody bevacizumab [3] and the PARP (poly (ADP-ribose) polymerase) inhibitors olaparib and rucaparib, which are FDA (Food and Drug Administration)-approved for platinum-sensitive recurrent BRCA-mutated ovarian cancer patients [4,5].

The most commonly reported gene mutations, highly associated with epithelial ovarian cancer, are for *TP53*, *BRCA1/2*, *PIK3CA* and *KRAS* genes [6]. The frequency of the above-mentioned mutations varies among the subtypes of epithelial ovarian cancer. Mutations in *TP53* are present in more than 96% of ovarian cancer cases [6,7]. *BRCA1/2* mutations are associated with the majority of hereditary ovarian cancer or Lynch syndrome and the mutation rate of *BRCA1/2* increases in recurrent HGSOC [6,7]. *PIK3CA* mutations have been also detected at high frequencies in ovarian clear cell carcinoma (OCCC) and endometrioid ovarian cancer related to endometriosis [6]. *NOTCH3* mutations have been detected in 66% of HGSOC cases and *NOTCH3* inactivation could be a potential therapeutic approach [7]. Low-grade serous ovarian carcinomas (LGSOCs) are associated with *BRAF* (especially V600E) and *KRAS* mutations [6,7,8]. LGSOCs present with a lower frequency of somatic *TP53* and *BRCA1/2* mutations and are not associated with germline *BRCA1/2* mutations [8]. *RAD51C* and *RAD51D* have been demonstrated to be inherited ovarian cancer predisposition genes with mutation carriers showing HGSOC [8]. In addition, the deleterious germline mutations BRP1 (BRCA1-interacting protein 1) are mainly associated with the high-grade serous epithelial subtype [8,9].

Liquid biopsy, now widely recognized as an important tool for the follow-up of cancer patients, is mainly based on the analysis of circulating tumor cells (CTCs) and circulating tumor DNA (ctDNA), which provide a source of diagnostic or/and prognostic markers. The clinical significance of CTCs and ctDNA in ovarian cancer has been investigated in many studies to date [1,10,11,12,13,14,15,16]. We have recently reported that *ESR1* is methylated in HGSOC patients, and that there is a statistically significant concordance between *ESR1* methylation in primary tumors and paired ctDNA [17]. Recently, many studies have investigated the mutation profile of ovarian cancer patients in plasma-cfDNA in many genes, such as *TP53*, *PIK3CA*, *KRAS*, *BRAC1*, *BRAC2* and *EGFR* [18,19,20,21,22,23].

*ESR1* mutations have emerged as a key mechanism of resistance to endocrine therapy in patients with ER-positive metastatic breast cancer [24] and their detection is now considered to be highly promising as a prognostic and predictive biomarker in this type of cancer [24,25]. To date, only a few studies have investigated the presence of *ESR1* mutations in endometrial and cervical cancer [26,27,28,29,30]. According to the cBioPortal cancer genomics database, *ESR1* mutations have been detected in 4–6% of uterine corpus endometrial carcinoma samples [31]. As for ovarian cancer, the cBioPortal cancer genomics database includes one ovarian serous cystadenocarcinoma study in which *ESR1* mutations were detected in 0.8% of samples [31]. In 2018, Stover et al., using targeted next-generation sequencing (NGS), detected a Y537S *ESR1* mutation in one patient with low-grade serous ovarian cancer (LGSOC); this particular patient developed a single site of progressive disease in an abdominal wall nodule and maintained stable low-volume peritoneal disease during endocrine therapy for almost five years, but later presented progressive disease after a durable response to hormonal therapy [32].

The aim of our study was to evaluate the frequency and the clinical relevance of *ESR1* mutations in HGSOC (HGSOC). We applied our recently developed highly sensitive and specific ESR1-NAPA assay for the detection of *ESR1* hotspot mutations (Y537S, Y537C, L536R, Y537N and D538G) [33] in combination with drop-off ddPCR [34] to investigate *ESR1* mutational status in primary tumors and plasma cfDNA in HGSOC patients.

## 2. Materials and Methods

### 2.1. Clinical Samples

The study material consisted of (a) primary formalin-fixed paraffin-embedded tumor tissues (FFPEs) from patients with HGSOC prior to any systemic treatment (*n* = 60) and, as a corresponding non-cancerous control, a group of 10 normal fallopian tube FFPEs that were obtained from women at the productive age; and (b) plasma-cfDNA samples from 80 patients with advanced (*n* = 20) and metastatic ovarian cancer (*n* = 60), and as a corresponding control, plasma-cfDNA samples from female healthy donors (HD, *n* = 11). All patients received at least six cycles of carboplatinum AUC 5 and paclitaxel at 175 mg/m^2^. Patients provided written informed consent to participate in the study, which was approved by the Local Essen Research Ethics Committee (16-6916-BO; 17-7859-BO), and the General University Hospital of Alexandroupolis’ ethics committee (date: 25 June 2020). The available clinicopathological features are shown in Table 1 and Table 2.

### 2.2. DNA Isolation

FFPEs: FFPEs containing >60% tumor cells were used for genomic DNA (gDNA) extraction. gDNA was isolated from FFPEs with using the QIAamp^®^ DNA FFPE Tissue Kit 50 (Qiagen^®^, Hilden, Germany), according to the manufacturer’s instructions. The DNA concentration was determined using a Nanodrop ND-1000 spectrophotometer (Nanodrop Technologies, Wilmington, NC, USA).

Plasma: 10 mL of peripheral blood in EDTA were used within 2–4 h to isolate plasma via centrifugation at 530× *g* for 10 min. Following a second centrifugation at 2000× *g* for 10 min, plasma was transferred into 2 mL tubes and stored at −70 °C until use. cfDNA was further isolated using the QIAamp Circulating Nucleic Acid Kit (Qiagen, Hilden, Germany), as previously described [35].

In all samples, cfDNA quality was checked prior to PCR using a previously described protocol [36]. Serial dilutions of a wild-type sample with a known DNA concentration (Human Reference DNA Female, Agilent Technologies, Santa Clara, CA, USA), prepared via serial 10-fold dilution in concentrations ranging from 200 ng/μL down to 0.5 ng/μL, were used to generate a standard curve for the quantification of the gDNA concentration in all cfDNA samples using a LightCycler z480 (Roche).

### 2.3. Drop-Off ddPCR for ESR1 Mutations (Y537S, Y537C, Y537N, L536R, D538G)

All cfDNA samples and controls were screened for *ESR1* mutations in exon 8, including the Y537S, Y537C, Y537N, D538G and L536R mutations, using drop-off ddPCR in a QX200 Droplet Digital PCR System (Bio-Rad Laboratories, Hercules, CA, USA), as previously described [34].

### 2.4. ESR1-NAPA Assay

All samples that were found to be positive for *ESR1* mutations via the *ESR1* drop-off ddPCR and all controls were further analyzed to define each individual *ESR1* mutation using our previously developed and validated ultrasensitive *ESR1*-NAPA assay for Y537S, Y537C, Y537N and D538G mutations [33]. Synthetic oligonucleotide sequences for each individual *ESR1* mutation were used as positive controls. In this study, we additionally designed, analytically validated and added the L536R mutation into our *ESR1*-NAPA assay. The experimental conditions for the *ESR1*-L536R mutation assay were optimized in detail regarding the annealing temperature, time and concentration of primers, buffer, MgCl_2_ (magnesium chloride solution), dNTPs (deoxyribonucleotide triphosphates) and BSA (bovine serum albumin solution) (data not shown).

### 2.5. Statistical Analysis

SPSS version 28.0 (IBM^®^ SPSS^®^ Statistics, Endicott, NK, USA) was used for statistical analysis. Pearson’s χ^2^ and Cohen’s kappa coefficient tests were used to estimate the concordance between *ESR1* mutations in primary tumors and paired cfDNA. The correlation between *ESR1* mutations and the clinicopathological characteristics of the patients (Table 1) were estimated using Pearson’s χ^2^ and Fischer’s exact test (*p*-values < 0.05 were considered statistically significant). Kaplan–Meier analysis was used for overall survival (OS) and progression-free survival (PFS) curves.

## 3. Results

**Figure 1:**
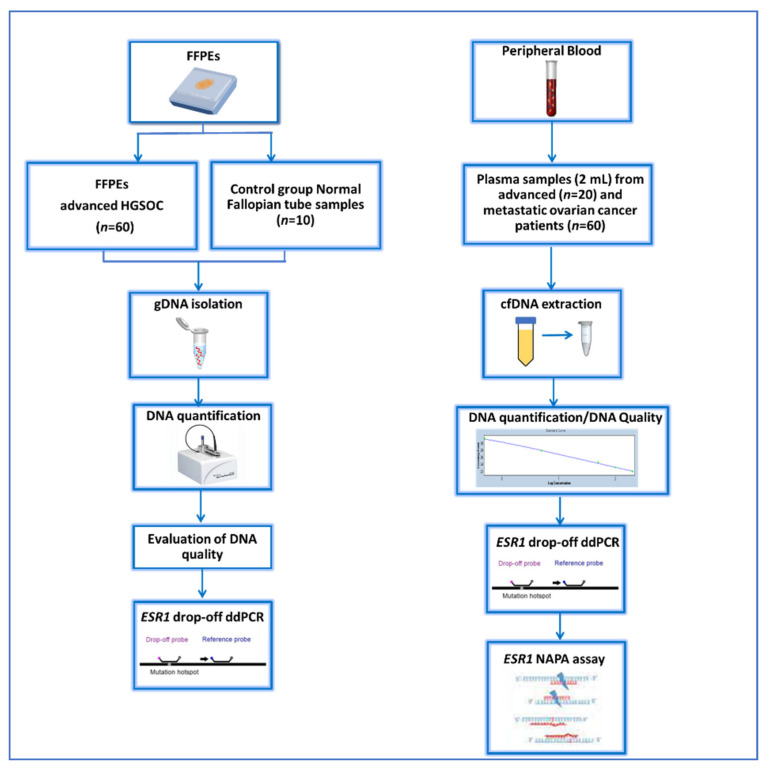
Schematic flowchart of the study.

A schematic flowchart of our study is given in Figure 1.

### 3.1. Detection of ESR1 Mutations in FFPEs

**Figure 2:**
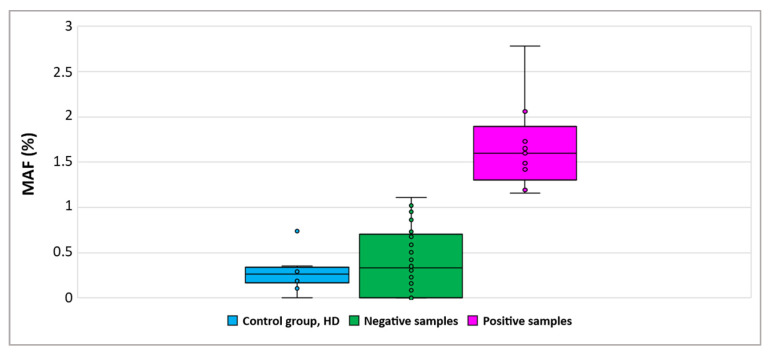
Detection of *ESR1* mutations in primary tumor samples using drop-off ddPCR. (MAF: mutant allele frequency).

To ensure the specificity of the drop-off ddPCR assay we first evaluated the mutant allelic frequency (MAF) in 10 non-cancerous fallopian tube samples. A cut-off value was calculated by adding the 2SD (standard deviation) to the mean of the MAF values of these control samples. The MAF% was estimated using the program developed by Attali et al. specifically for this type of ddPCR assay [37]. Based on the defined cut-off (1.15), we detected the presence of *ESR1* mutations in 9/60 (15%) of FFPE samples tested (Figure 2).

In this patient group the median PFS was 41 months, and the median OS was 47 months. There was no significant correlation between OS, PFS, and *ESR1* mutations in FFPEs when our results were evaluated via Kaplan–Meier analysis (data not shown). Furthermore, no significant correlation between *ESR1* mutations and the patients’ clinicopathological characteristics was observed.

### 3.2. Detection of ESR1 Mutations in Plasma-cfDNA

**Figure 3:**
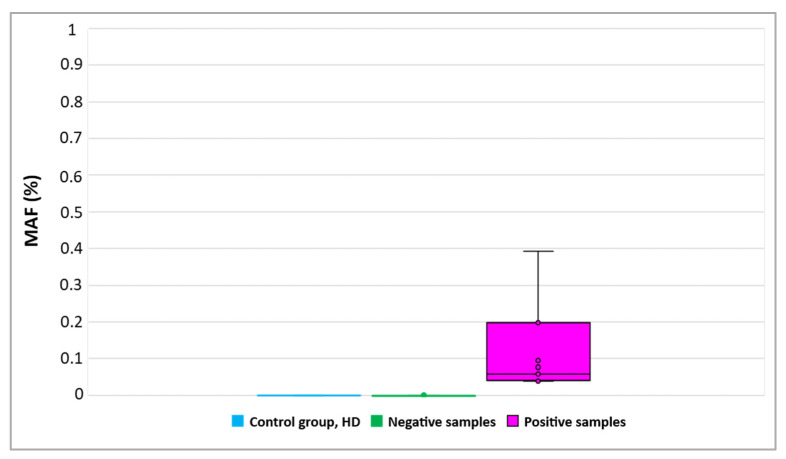
Detection of *ESR1* mutations in plasma-cfDNA samples using drop-off ddPCR. (MAF: mutant allele frequency).

**Figure 4:**
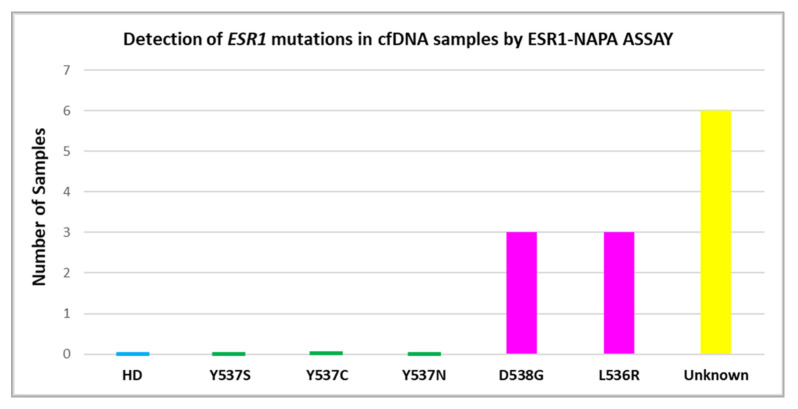
Detection of *ESR1* mutations in plasma-cfDNA samples using the NAPA-ESR1 assay.

Using drop-off ddPCR, *ESR1* mutations were detected in 11/80 (13.8%) plasma-cfDNA samples (Figure 3), more specifically, in eight plasma-cfDNA samples from patients with metastatic ovarian cancer (8/60, 13.3%) and in three plasma-cfDNA samples from patients with advanced ovarian cancer (3/20, 15%). All these *ESR1*-mutation-positive samples were further analyzed to define *ESR1* mutations using the *ESR1*-NAPA assay (Figure 4). The D538G mutation was detected in three plasma-cfDNA samples from patients with metastatic ovarian cancer and L536R was detected in two plasma-cfDNA samples from patients with metastatic ovarian cancer and in one plasma-cfDNA sample from one patient with advanced ovarian cancer. It should be mentioned that in one patient with metastatic ovarian cancer, both D538G and L536R were detected in plasma-cfDNA.

The median PFS was 38 months and the median OS was 38 months in the group of *n* = 20 patients with advanced ovarian cancer, and the median PFS was 19 months and the median OS was 31 months in the group of *n* = 60 patients with metastatic ovarian cancer. Kaplan–Meier analysis was performed to estimate the correlation between OS and PFS with the detection of *ESR1* mutations in both groups. No significant correlations were observed among OS, PFS and *ESR1* mutations for both groups (data not shown). Furthermore, no significant correlations between *ESR1* mutations and the patients’ clinicopathological characteristics were observed.

## 4. Discussion

We report, for the first time, the detection of *ESR1* mutations in primary tumors (FFPEs) in plasma cfDNA samples from patients with advanced and metastatic ovarian cancer patients using highly sensitive and specific methodologies based on drop-off ddPCR for screening and the *ESR1*-NAPA assay for the definition of Y537S, Y537C, Y537N, L536R and D538G *ESR1* mutations.

To date, the detection of *ESR1* mutations has been reported in cervical squamous cell carcinoma [26] and in a patient with endometrial cancer treated with an aromatase inhibitor [27]. It has also been reported that the presence of *ESR1* mutations is associated with worse outcomes in endometrial cancer [28]. Apart from endometrial cancer studies, there are very few studies that show the existence of *ESR1* mutations in ovarian cancer. More specifically, in 2017, McIntyre et al. detected *ESR1* Y537S mutation in one patient with low-grade serous ovarian carcinoma, when analyzing 26 primary tumor samples using NGS [38]. In 2018, Stover et al., using targeted NGS, detected a Y537S *ESR1* mutation in one patient with LGSOC; this particular patient developed a single site of progressive disease in an abdominal wall nodule and maintained stable low-volume peritoneal disease during endocrine therapy for almost five years, but later presented progressive disease after a durable response to hormonal therapy [32]. In 2019, Gaillard et al. reported that *ESR1* mutations were detected in 4.4% (24/548) of uterine endometrioid carcinomas vs. 0.2% (1/446) of uterine serous carcinomas and 3.5% (5/144) of ovarian endometrioid carcinomas compared to 0.3% (12/3502) of ovarian serous carcinomas, whereas in an ovarian serous carcinoma both *ESR1* Y537S and D538G mutations were detected [39]. Since then, there have been no reports on the detection of *ESR1* mutations in ovarian cancer.

In the present study, we report that, using drop-off ddPCR, *ESR1* mutations were detected in 15% of primary tumor tissues and in 13.8% of plasma-cfDNA samples tested. More specifically, eight plasma-cfDNA samples from patients with metastatic cancer and three plasma-cfDNA samples from patients with advanced ovarian cancer were found to be positive for *ESR1* mutations. All plasma-cfDNA samples found to be positive via *ESR1* drop-off ddPCR were further analyzed using the *ESR1*-NAPA assay in order to define the specific mutation. In patients with metastatic ovarian cancer, the D538G mutation was detected in three plasma-cfDNA samples and L536R was detected in two plasma-cfDNA samples, whereas both D538G and L536R were detected in one patient. In patients with advanced ovarian cancer, L536R was detected only in one plasma-cfDNA sample. Drop-off ddPCR screens for *ESR1* mutations were clustered in exon 8. Hence, any mutation in this region could be detected in addition to Y537S, Y537C, Y537N, L536R and D538G. In this region, additional mutations were present, such as L536H, which has been detected in endometrial cancer [29,39].

These findings could be of clinical importance if we consider that in metastatic breast cancer the detection of *ESR1* mutations has led to the development of novel highly promising therapeutic strategies. In patients with metastatic breast cancer that are positive for *ESR1* mutations, selective estrogen receptor modulators (SERMs) and selective estrogen receptor covalent antagonists (SERCAs) are now being evaluated as promising drugs. Lasofoxifene is currently in Phase 2 trials for patients with *ESR1* mutations and for patients after progression on endocrine therapy and CDK4/6 inhibition [40]. The FDA has granted a fast-track designation to lasofoxifene for use as a treatment of female patients with estrogen receptor (ER)-positive, HER2-negative metastatic breast cancer who harbor *ESR1* mutations. Bazedoxifene, a SERM/SERD hybrid, which has been approved for use in postmenopausal hot flashes and osteoporosis, is now in a Phase 2 trial for patients after progression on endocrine therapy (NCT02448771) [40]. Fanning et al. reported that bazedoxifene possessed improved inhibitory potency against the Y537S and D538G mutants compared to tamoxifen and had additional inhibitory activity in combination with the CDK4/6 inhibitor palbociclib [41]. In parallel, the efficacy of H3B-6545, a drug optimized from the SERCA class, against *ESR1* mutations was demonstrated in patients with metastatic breast cancer previously treated with endocrine therapy and CDK4/6i [42]. H3B-6545 is now in a Phase 2 trial for patients after progression on endocrine therapy and CDK4/6i (NCT03250676) [40]. The combined analysis of SoFEA and EFFECT showed that patients with *ESR1* mutations detected in plasma-cfDNA samples [43] had shorter PFS and OS when treated with exemestane therapy, compared with fulvestrant. In the PALOMA-3 trial, patients on fulvestrant and a placebo tended to have poorer PFS in the presence of mutations compared to the absence of mutations [44]. O’Leary et al. reported that *ESR1* Y537S mutation promotes resistance to fulvestrant and that acquired mutations from fulvestrant are a major driver of resistance to fulvestrant and palbociclib combination therapy [45]. In the phase 3 PADA-1 trial presented at the 2021 San Antonio Breast Cancer Symposium, it was observed that when switching from an aromatase inhibitor plus palbociclib to fulvestrant and palbociclib upon early identification of the *ESR1* mutation in plasma—before disease progression—the median PFS was doubled. This trial has also shown that *ESR1* mutations are rarely detected in the plasma-cfDNA of ER + HER2− metastatic breast cancer patients with no overt resistance to aromatase inhibitors and that the detection of *ESR1* mutations was associated with a significantly shorter PFS, suggesting that the presence of the *ESR1* mutation at baseline could accelerate the outset of resistance to AI-palbociclib [46,47]. Novel therapies could include possible strategies to overcome the endocrine resistance induced by *ESR1* mutations.

## 5. Conclusions

To our knowledge, this is the first time that the presence of *ESR1* mutations has been reported in primary tumors and plasma-cfDNA from HGSOC patients. The clinical significance of this finding should be examined prospectively in a large group of ovarian cancer patients.

## Figures and Tables

**Table 1:** Clinicopathological characteristics of the advanced HGSOC patients.

Clinicopathological Characteristics	Primary FFPE Tumor Tissues (Total *n* = 60) *n*%
Histology	
Serous	60 (100)
Tumor grade (G)	
G1	2 (3.3)
G2	26 (43.3)
G3	32 (53.4)
FIGO stage	
I	4 (6.7)
II	2 (3.3)
III	46 (76.7)
IV	8 (13.3)
Age	Median age = 63
≥median age	30 (50.0)
<median age	30 (50.0)
Distant metastasis (M)	
M0	51 (85.0)
M1	8 (13.3)
Unknown	1 (1.7)

**Table 2:** Clinicopathological characteristics of the advanced and metastatic ovarian cancer patients.

Clinicopathological Characteristics	Plasma-ctDNA Samples (Total *n* = 80) *n*%
Histology	
Serous	80 (100)
Tumor grade (G)	
G1	28
G2-G3	43
Unknown	7
FIGO stage	
I	-
II	11 (13.75)
III	8 (10.00)
IV	41 (51.25)
Unknown	20 (25.00)
Age	Median age = 62
≥median age	41 (51.25)
<median age	39 (48.75)
Metastasis (M)	
M0	20 (25.00)
M1	58 (72.50)
Unknown	2 (2.50)
Therapy	
Docetaxel/Carboplatin	35 (43.75)
Carboplatin	4 (5.00)
Docetaxel	2 (2.50)
Docetaxel/Carboplatin/Avastin	18 (22.5)
Docetaxel/Avastin	2 (2.50)
Paclitaxel (Taxol)/Carboplatin/Avastin	1 (1.25)
Paclitaxel (Ovapac, Taxol, Taxoprol)/Carboplatin	8 (10.00)
Oxaliplatin/Capecitabine	1 (1.30)
Unknown	8 (10.00)

## Data Availability

The data presented in this study are available on request from the corresponding author. The data are not publicly available due to ethical restrictions.
